# Bifunctional Photocatalysts: Exploiting Proximity for Enhanced Reaction Performance

**DOI:** 10.1002/chem.70842

**Published:** 2026-03-04

**Authors:** Luigi Dolcini, Daniele Lavelli, Alberto Dal Corso, Luca Pignataro

**Affiliations:** ^1^ Dipartimento di Chimica Università degli Studi di Milano Milano Italy; ^2^ Fakultät für Chemie und Pharmazie Universität Regensburg Regenbsurg Germany

**Keywords:** bifunctional photocatalyst, cooperative effects, cross‐coupling, cycloaddition, photocatalysis

## Abstract

The bifunctional approach has often been employed in catalysis to obtain more effective and enantioselective transformations. In the sub‐area of photocatalysis, bifunctional systems have been designed (i) to introduce chirality in the system and thus gain stereocontrol, and (ii) to more effectively exploit the short‐lived catalytic intermediates (e.g., photoexcited species and radicals). In some cases, instead of two distinct catalytic units, a single photoactive group displaying also other types of activity can be employed (“bivalent photocatalysts”). This review aims to cover the most recent examples in this field, establishing—when possible—a comparison with the corresponding dual catalytic systems.

## Graphical abstract

This review covers the application of the bifunctional approach to photocatalysis as a means to attain (enhanced) enantioselectivity, and, more in general, as a strategy to enhance the catalytic performance through an effective use of short‐lived reaction intermediates.

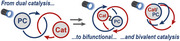

## 1 Introduction

**FIGURE 1:**
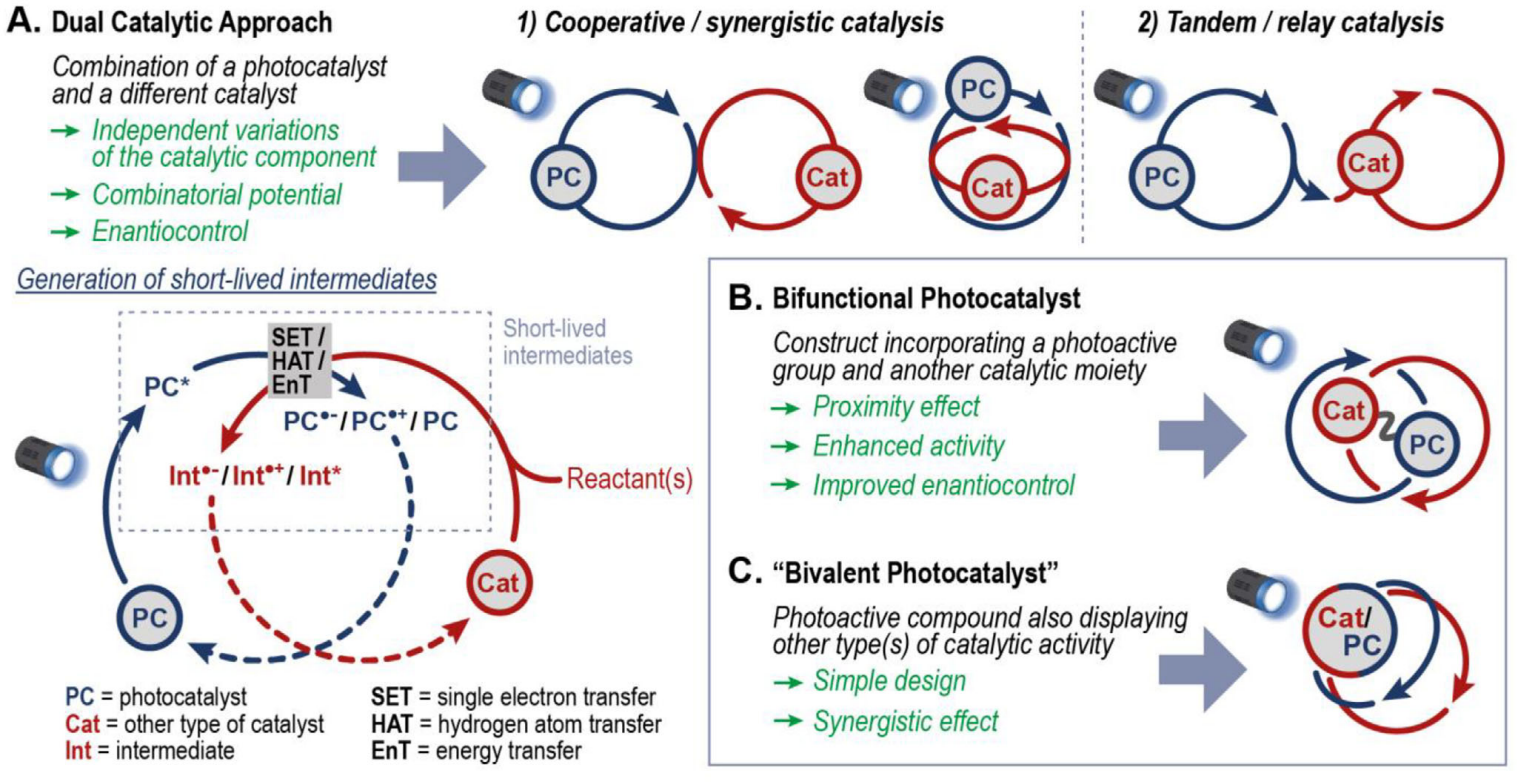
Dual catalytic approach and possible limitations associated to the generation of short‐lived intermediates (A). Bifunctional (B) and “bivalent photocatalysts” (C) as possible improvement.

Following up on the pioneering article by Nicewicz and MacMillan [1], the last two decades have seen a tremendous development of visible light‐promoted synthetic methodologies [2, 3, 4, 5, 6, 7]. With quite basic reaction setups and mild conditions, visible light may provide access to radical intermediates, thus disclosing reaction manifolds not accessible with “traditional” ionic chemistry. As the majority of organic compounds do not absorb visible light, a photocatalyst (PC) is required in most instances. Upon photoexcitation, the latter (PC*) becomes able to perform photoinduced electron transfer (PET) or energy transfer (EnT) onto the reactant(s). Since visible light can be regarded as a mild and traceless reactant, photocatalytic cycles have been successfully combined with other types of catalytic manifolds [8, 9, 10], such as organocatalysis [1, 11] and transition metal catalysis [12, 13, 14, 15]. In dual catalytic systems (Figure 1A), the PC and the other catalyst(s) may act either synergistically (i.e., taking part in the same catalytic cycle) or according to a tandem/relay scheme in which they operate independently (e.g., the photocatalytic cycle can generate the catalyst for a different cycle). The dual catalytic approach (Figure 1A) has the advantage of a “combinatorial” reaction optimization, because the catalytic components can be varied independently. However, considering that photoexcited species PC* and other catalytic cycle intermediates (such as radicals, radical anions/cations – Figure 1A) are generally short‐lived (e.g., PC* lifetime typically ranges from 1–2 µs to a few ns) [16, 17, 18, 19], enforcing proximity of the photoactive moiety with other catalytic group(s) may bring remarkable improvements, such as: (a) more effective use of short‐lived intermediates, which don't need to diffuse through the solution to meet the reaction partner [20]; (b) substrate binding/activation; (c) effective transfer of the stereochemical information possibly present in the catalyst. Thus, a carefully designed “bifunctional photocatalyst” (Figure 1B) may repay for the additional synthetic effort it requires compared to the corresponding dual catalytic system. The Dixon's definition of a bifunctional catalyst as a “*low molecular weight, structurally defined molecule possessing two distinct functional groups to bring about new reactivity and/or selectivity in a reaction of interest*” [21] covers a large share of organocatalysts [22, 23, 24, 25], as well as several transition metal complexes [26, 27, 28, 29, 30, 31, 32]. Recently, in a context of increasing interest for visible light‐promoted methodologies, the bifunctional approach has been extended also to photocatalysis, and the number of reported examples is rapidly growing, including a few cases in which non‐covalent interactions are employed to assemble the bifunctional construct.

This review covers the area of bifunctional photocatalysis (Figure 1B) with particular emphasis on the most recent reports. After an overview on chiral catalysts (which have been previously surveyed elsewhere [33, 34]), examples will be discussed in which bifunctionality allows to obtain enhanced catalytic performance with respect to already established dual catalytic systems. The last section surveys systems that address two different catalytic tasks with a single photoactive group (Figure 1C). Since, according to the above‐mentioned definition, such PCs cannot be defined as “bifunctional”, herein they are called “bivalent photocatalysts”.

## 2 The Bifunctional Approach in Photocatalysis

### 2.1 Enantioselective Systems

**SCHEME 1:**
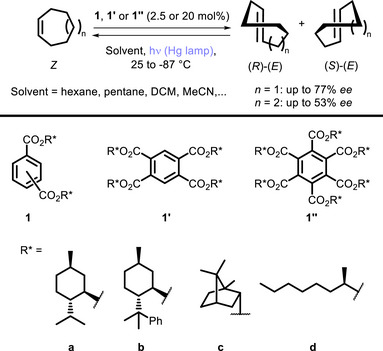
Sensitized isomerization of cycloheptene and cyclooctene.

The use of chiral photosensitizers to perform enantioselective transformations has been studied by photochemists since the 1970s [35]. Inoue and co‐workers reported some of the most successful examples in the field, with the enantioselective photoisomerization of *Z*‐ to *E*‐cyclooctene [36, 37, 38, 39] and cycloheptene [40] using different chiral esters (**1a‐d**, Scheme 1). The effect of pressure, temperature, and irradiation time is shown to have some effect on the observed enantiomeric excess.

These examples demonstrated the possibility of using chiral photosensitizers to activate the substrates while also controlling the stereochemical outcome of the reaction, but the enantioselectivity remained moderate at best.

**SCHEME 2:**
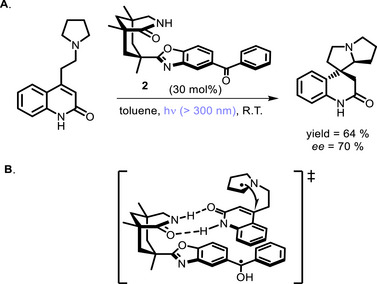
Pyrrolizidine cyclization with bifunctional PC **2** (A) and proposed transition state (B).

In 2002, the ‘bifunctional approach’ was brought into the field by the pioneering work of Bach and co‐workers, who firstly reported the use of photoactive chiral hosts derived from Kemp's triacid to hydrogen‐bind the substrate and attain highly enantioselective *intramolecular* photocyclizations [41]. While stoichiometric chiral hosts were initially used [42], benzophenone‐derived catalysts (**2**) were soon employed in catalytic amounts (2005) to promote the cyclization of spirocyclic pyrrolizidines (Scheme 2A) [43]. In these systems, the lactam group is responsible for substrate binding and positioning in a chiral environment, whereas the benzophenone moiety has the role of absorbing light and generating the α‐amino radical, which then undergoes cyclization with up to 70% *ee* (Scheme 2B).

**SCHEME 3:**
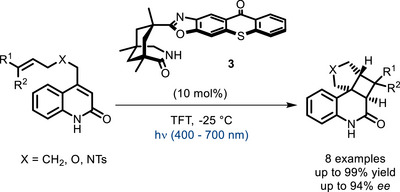
Enantioselective intramolecular [2+2] cycloaddition catalyzed by a bifunctional thioxanthone derivative. TFT = trifluorotoluene.

Some years later, in 2014, the same group reported the use of a bifunctional thioxanthone‐derived PC (**3**) to increase the stereoselectivity in an intramolecular [2+2] photocycloaddition [44]. As in the previous work, the Kemp's triacid‐derived moiety was used as a hydrogen bond donor/acceptor, capable of coordinating the substrate and creating an enantioface discrimination. The thioxanthone moiety acts as the photosensitizer, and the energy absorbed from light is then delivered to the substrate, probably via triplet‐energy transfer, triggering the subsequent cyclization (Scheme 3). The same kind of reactivity was later extended to 3‐alkyl‐4‐alkenyloxyquinolones [45].

**SCHEME 4:**
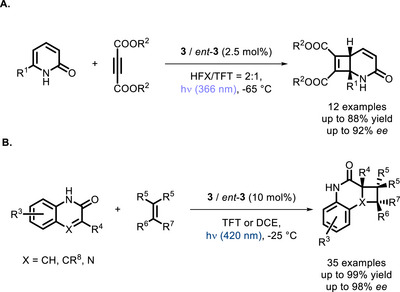
Enantioselective intermolecular [2+2] cycloaddition with substituted alkynes (A) and alkenes (B) catalyzed by a bifunctional thioxanthone derivative. HFX = hexafluoro‐*m*‐xylene. TFT = trifluorotoluene.

Kemp's triacid‐derived bifunctional PCs were also successfully applied to the more challenging enantioselective [2+2] *intermolecular* photocycloadditions. In 2014, shortly following a ground‐breaking report by Yoon and co‐workers on a dual catalytic approach (chiral Lewis acid + Ru‐based PC) to one such difficult transformation [46], Bach and co‐workers reported the enantioselective [2+2] cycloaddition between alkynes and pyridines using the bifunctional thioxanthone derivative **3** or its enantiomer *ent*‐**3** (Scheme 4A) [47]. Some years later, the Bach group used the same approach to carry out the cycloaddition of activated alkenes to quinolones [48], and then to quinoxalines and alkenes (Scheme 4B) [49].

**SCHEME 5:**
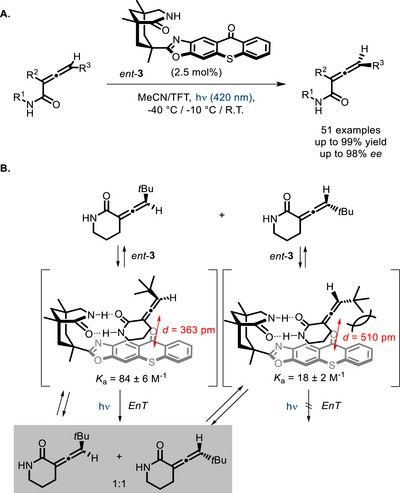
Deracemization of chiral allenes sensitized by a bifunctional thioxanthone derivative (A), and proposed mechanism (B).; TFT = trifluorotoluene.

Another class of reactions in which these bifunctional PCs are effective is deracemization reactions. The first successful example was reported in 2018 by the Bach group on allenes [50]. After engaging in hydrogen bonding with the substrate, the PC *ent*‐**3** acts as a triplet energy sensitizer toward the enantiomeric form, which binds at a shorter distance from the thioxanthone group (Scheme 5), whereas sensitization of the other enantiomer is inefficient. The achiral triplet state evolves into the racemic mixture of allenes, and this leads to progressive accumulation of the enantiomer not undergoing photosensitization. The role of Kemp's triacid‐derived lactam moiety was demonstrated by measuring the association constants between the PC and the substrate by NMR titration. Catalyst **3**/*ent*‐**3** proved also effective in the deracemization of different allenes [51, 52] and of certain alkenes [53], provided that they possess an amide recognition unit for binding the bifunctional PC.

**SCHEME 6:**
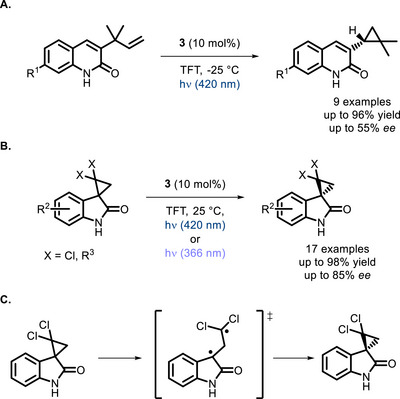
Formation of chiral cyclopropanes from alkenes (A), deracemization of chiral cyclopropanes (B), and deracemization mechanism through formation of 1,3‐diradicals (C). TFT = trifluorotoluene.

The same kind of approach (and catalyst) allowed to achieve the enantioselective formation of chiral cyclopropanes [54] through a di‐π‐methane rearrangement [55] (Scheme 6A), as well as their deracemization (Scheme 6B) [56]. The formation of a short‐lived 1,3‐diradical, formed from the photosensitized opening of the 3‐membered ring, was proposed and confirmed by transient absorption spectroscopy measurements (Scheme 6C).

**SCHEME 7:**
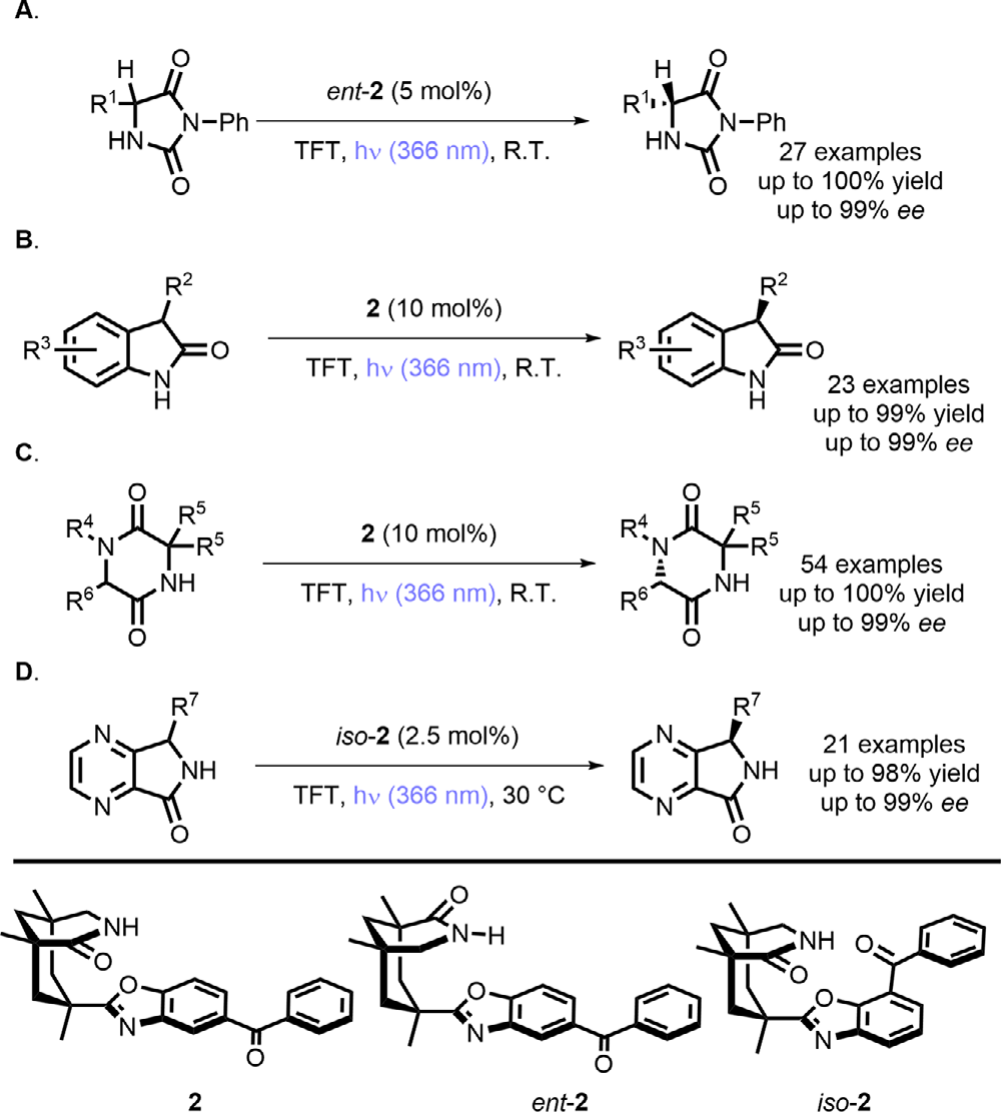
Deracemization of hydantoins (A), oxindoles (B), 2,5‐diketopiperazines (C) and 4,7‐diazaisoindolines (D). TFT = trifluorotoluene.

In 2021 and in the following years, Bach reported that benzophenone derivatives (**2**, *ent*‐**2,** and *iso*‐**2**) can be used for the photochemical deracemization of hydantoins (Scheme 7A) [57], oxindoles (Scheme 7B) [58], 2,5‐diketopiperazines (Scheme 7C) [59], and 4,7‐diazaisoindolinones (Scheme 7D) [60].

**SCHEME 8:**
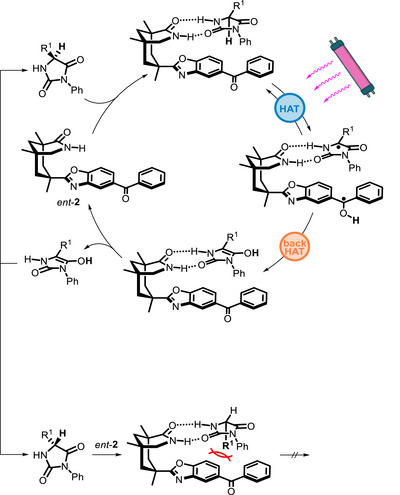
Catalytic cycle for the deracemization of hydantoins.

In these reactions, enantiodiscrimination stems from the ability of the PC to racemize one substrate's enantiomer through a HAT/back HAT cycle (generating the enol form), without affecting the other enantiomer (Scheme 8). In this way, the “unreactive” enantiomer is progressively accumulated at the expense of the reactive one.

Finally, the Kemp's triacid‐derived motif was also used bound to Ru^II^ porphyrins and to Ir^III^ polypyridyl complexes to achieve, respectively, asymmetric oxygenation reactions [61] and enantioselective rearrangements [62].

**SCHEME 9:**
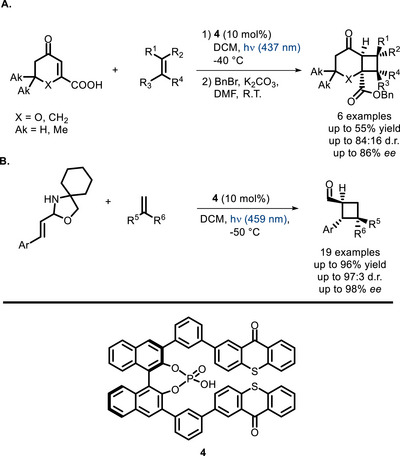
[2+2] Enantioselective photocycloaddition of alkenes and carboxylic acids (A) and alkenes and *N*,*O*‐acetals derived from cinnamic aldehydes (B) catalyzed by a bifunctional phosphoric acid derivative.

Another important class of bifunctional PCs is represented by phosphoric acid derivatives, deriving from conjugation of these popular organocatalysts [63] to suitable photoactive units. In 2020, Bach and co‐workers reported the bifunctional catalyst **4** (Scheme 9)—a chiral BINOL‐derived phosphoric acid possessing two thioxanthone photoactive units [64]. This catalyst was found to promote the [2+2] photocycloaddition of β‐carboxy‐substituted cyclic enones to alkenes (Scheme 9A) with good enantioselectivity and better efficiency compared to the parent PC (thioxanthone). Luminescence measurements showed that **4** has remarkably lower triplet energy than thioxanthone, and formation of strong hydrogen bonds between the (P = O)OH moiety and the substrate's COOH group was confirmed by DOSY NMR studies. These findings led the authors to hypothesize that coordination to the catalyst by hydrogen bonding with its carboxy group probably decreases the enone's triplet energy, analogously to what had been reported for simple enones in the presence of Lewis acids [65, 66]. In another contribution, PC **4** is used to promote highly enantioselective [2+2] cycloadditions of cinnamaldehyde‐derived *N*,*O*‐acetals to alkenes (Scheme 9B) [67]. The reaction occurs with very high enantioface discrimination, seemingly deriving from the complexation of the phosphoric acid with the substrate.

Masson and co‐workers developed other bifunctional PCs of this kind, combining BINOL‐derived phosphoric acids with different moieties capable of absorbing light [68], which were tested in the asymmetric electrophilic amination of α‐unsubstituted enecarbamates. Similarly, the group of Takagi showed the application of a BINOL‐derived phosphoric acid bearing only one thioxanthyl moiety in the [2+2] cycloaddition of quinolones [69].

**SCHEME 10:**
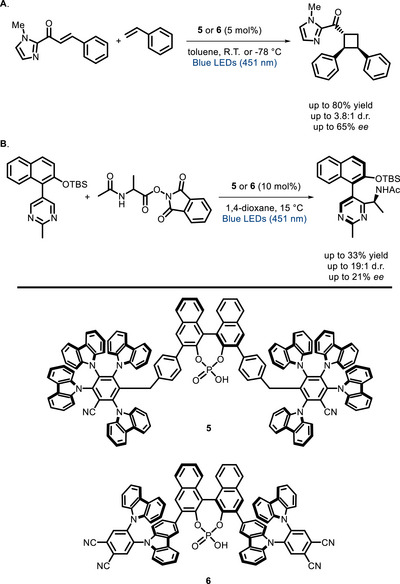
[2+2] cycloaddition reaction tested with bifunctional PCs **5** and **6** (A). Minisci reaction tested with PCs **5** and **6** (B).

As one of the recent contributions to this field, König and Toste were able to develop BINOL‐derived phosphoric acids (**5** and **6** in Scheme 10) where the PC moiety consists of a donor‐acceptor (D‐A) cyanoarene unit [70] This work is among the first contributions where this versatile class of dyes [18, 71, 72, 73, 74, 75, 76, 77] is used to develop a bifunctional PC. These catalysts were tested in two visible light‐promoted stereoselective reactions: i) a [2+2] cycloaddition of *C*‐cinnamoyl imidazole to styrene originally reported by Yoon and co‐workers (Scheme 10A) [78]; ii) a Minisci‐type coupling of 2‐methyl‐5‐naphthylpyrimidines to α‐aminoacid *N*‐hydroxyphthalimide esters (Scheme 10B) [79]. In these proof‐of‐concept reactions, PC **5** and **6** showed promising levels of activity and stereoselectivity, yet not reaching the performance level of the original dual catalytic reactions [78, 79].

**SCHEME 11:**
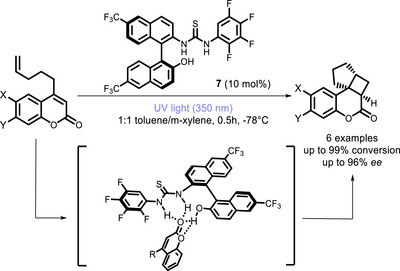
Intramolecular [2+2] photocycloaddition of 4‐alkenylcoumarin catalyzed by bifunctional thioureas.

**SCHEME 12:**
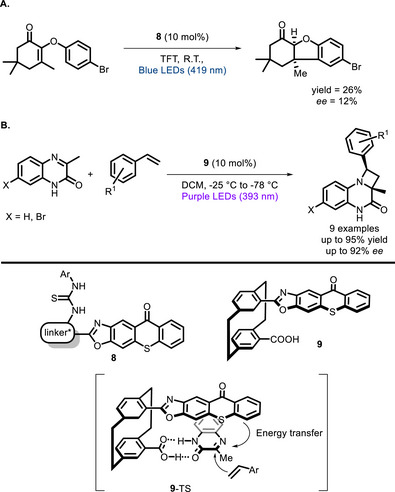
Thiourea‐bearing PCs developed by Bach (A) and Hong (B), and their application in stereoselective cycloadditions.TFT = trifluorotoluene.

The thiourea group is another functionality that has been widely applied in organocatalysis to attain orientation and activation of substrates possessing donor atoms [80, 81], and applications to photocatalysis have started appearing in the literature. The group of Sibi and Sivaguru developed a near UV‐promoted intramolecular [2+2] photocycloaddition of 4‐alkenylcoumarin catalyzed by various chiral thioureas featuring a BINOL moiety, the best performances being achieved with **7** [82]. The key to the observed enantioselectivity lies within the hydrogen bonding interactions between the thiourea moiety of the catalyst and the carbonyl functionality of the substrate, which ensures a highly selective reaction pathway (Scheme 11). Additionally, the study revealed that while both the substrate and sensitizer are capable of efficiently absorbing light, the reaction proceeds at a significantly slower rate in the absence of the chiral catalyst. This suggests that the catalyst not only dictates enantioselectivity but also plays a crucial role in enhancing the overall reaction kinetics. Bifunctional thiourea PCs (**8**) were also synthesized by Bach and co‐workers (Scheme 12A) and preliminarily tested in visible light‐promoted photocyclizations [83].

Among the hydrogen bond‐donor bifunctional PCs, it is worth mentioning also the work of Hong and co‐workers, who developed [2.2]paracyclophane PCs (**9**) featuring the free COOH as the hydrogen bond donor group (Scheme 12B). Catalyst **9** was successfully applied to the Paternò‐Büchi reaction between 3‐methylquinoxalin‐2(1*H*)‐one and styrenes [84], which occurred with good enantiomeric excesses owing to substrate binding into a chiral pocket.

**SCHEME 13:**
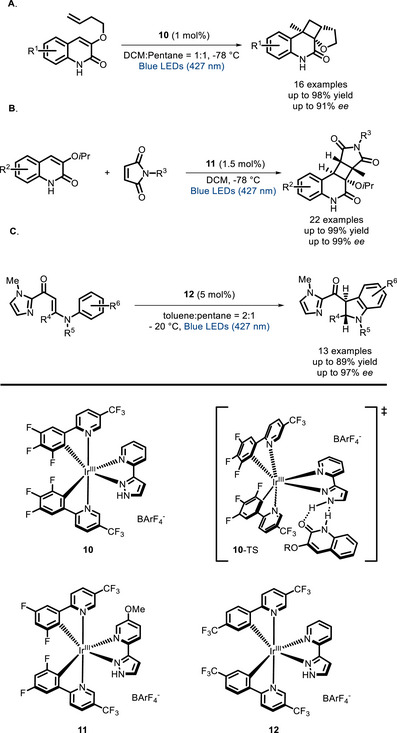
[2+2] cycloadditions and 6π photoelectrocyclization catalyzed by chiral Ir^III^ complexes, and the transition state. BArF_4_ = tetrakis(3,5‐bis(trifluoromethyl)phenyl)borate.

The group of Yoon implemented the bifunctional approach on classical Ir^III^ PCs, synthesizing bifunctional Ir^III^ complexes (**10**, **11,** and **12**) featuring one ligand able to interact with the substrate by hydrogen bonding (**10**‐TS). In this case, the stereochemical information resides within the photoactive moiety, that is, the chiral helical Ir^III^ complex. These complexes proved to be successful in different enantioselective transformations, such as intra‐ [85] (Scheme 13A) and intermolecular [2+2] cycloadditions (Scheme 13B) [86], and 6π photoelectrocyclizations (Scheme 13C) [87].

**SCHEME 14:**
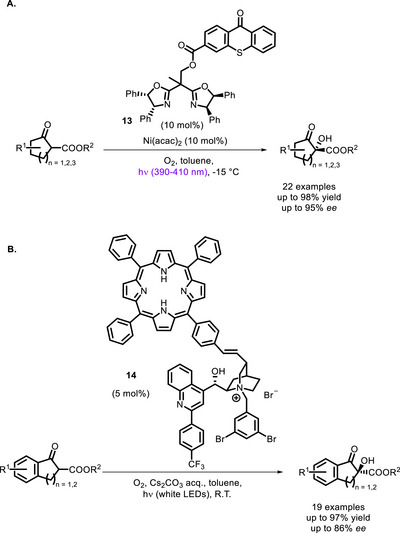
Aerobic oxidation of β‐ketoesters catalyzed by a Ni‐BOX bifunctional complex (A) and a PTC‐porphyrin system (B).

Xiao and co‐workers linked several chiral bis‐oxazoline ligands with a thioxanthone residue. The corresponding in situ‐formed Ni‐complexes were employed to catalyze the enantioselective aerobic oxidation of β‐ketoesters (the best performing PC, **13**, is shown in Scheme 14A) [88]. Later on, Meng and colleagues reported similar visible light‐promoted aerobic oxidations employing bifunctional PCs consisting of a tetraphenylporphyrin photosensitizer connected to a cinchona‐derived phase‐transfer catalyst (PTC). The best performing PC (**14** in Scheme 14B) showed high catalytic activity and a good level of stereocontrol [89].

**SCHEME 15:**
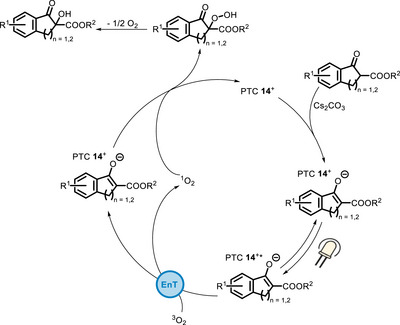
Catalytic cycle for the reaction involving the bifunctional PTC **14**.

In these systems, while the **13**‐Ni^II^ complex and the PTC **14** act as Lewis acids, binding the enolate of the ketoester and keeping it in a chiral environment, the thioxanthone/porphyrin moiety sensitize the formation of singlet oxygen, which can then react with the enolate (Scheme 15).

### 2.2 Bifunctional Systems Displaying Increased Catalytic Activity

As mentioned in the introduction, the bifunctional approach represents a strategy to achieve a better integration between the catalytic cycles employed, thus reaching enhanced catalytic performance. While in Section 2.1 the focus was on the chiral enantioselective systems, this section describes examples in which it is mainly the catalytic activity that benefits from bifunctionality. The underlying idea is that keeping the short‐lived intermediates of photocatalyzed reactions (e.g., excited states, radical anions/cations) close to the substrate can allow to exploit them more effectively than in the corresponding dual catalytic reactions.

**FIGURE 2:**
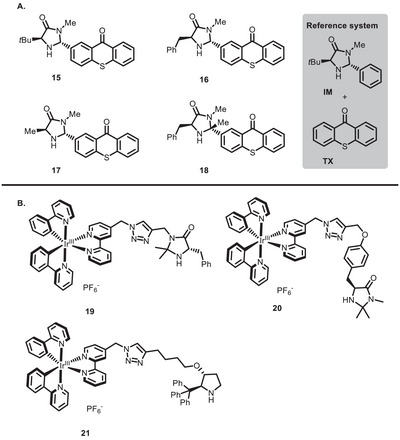
Bifunctional PCs synthesized by Alemán (A) and Cozzi (B).

**SCHEME 16:**
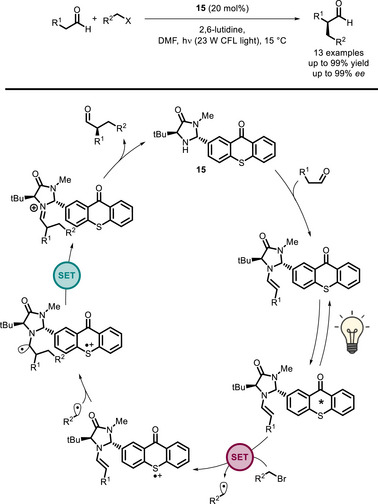
Proposed catalytic cycle of the enantioselective α‐alkylation of aldehydes promoted by bifunctional PC **15**.

One of the first examples in this sense was reported by Alemán and co‐workers in 2018 [90]. In this work, a series of imidazolidinone catalysts functionalized with a thioxanthone moiety (**15**‐**18**, Figure 2A) were synthesized and found able to successfully catalyze the enantioselective α‐alkylation of aldehydes (Scheme 16) [91]. These bifunctional PCs gave slightly better yields and *ee*s with respect to the reference dual system employing a thioxanthone (**TX**) and imidazolidinone **IM** (Figure 2A). Although mechanistic investigation hinted that the improvement in the yields and *ee*s might be mostly due to the increased steric hindrance, the importance of this study was to highlight the effect of having the PC covalently bound to the imidazolidinone. Indeed, it was demonstrated that the contribution by the alkyl bromide‐enamine electron donor‐acceptor (EDA) complex [92] or by the enamine itself [93] to initiate the catalytic cycle was negligible, meaning that the thioxanthone moiety was playing an active role in catalysis. Further studies about the quantum yield (Φ) of the **15**‐catalyzed reaction showed that a chain mechanism operates (Φ > 1), but with remarkably lower quantum yield than in the dual catalytic reaction (reference system in Figure 2A) [94, 95]. From this finding, it can be inferred that reaction initiation by alkyl bromide reduction occurs by oxidative quenching of the thioxanthone moiety (Scheme 16) to a larger extent than in the corresponding dual catalytic system.

In 2020, Gualandi, Ceroni, Lombardo, Cozzi, and co‐workers reported three bifunctional imidazolidinone‐conjugated Ir^III^ PCs (**19**‐**21**, Figure 2B), which showed significant yield improvement in the α‐alkylation of aldehydes compared to the corresponding dual catalytic systems [96], seemingly due to proximity between the aminocatalyst and the photoactive unit.

**SCHEME 17:**
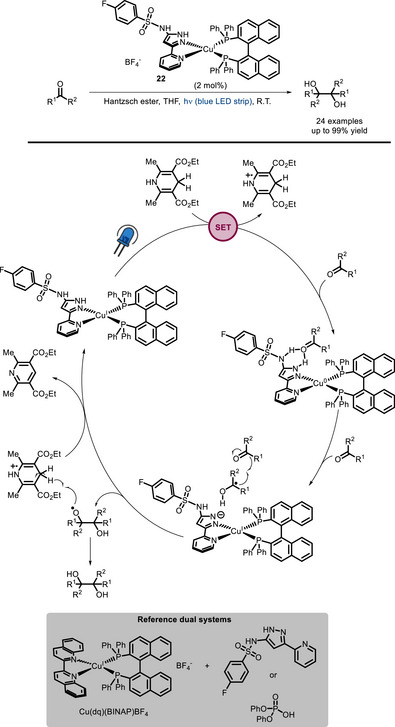
Pinacol coupling catalyzed by bifunctional Cu complex **22**.

In 2019, a bifunctional Cu‐based PC (**22**) for pinacol‐type reductive couplings was reported by Collins and co‐workers using the Hantzsch ester as a terminal reductant [97]. By functionalizing the Cu^I^‐binding pyrazole‐pyridine ligand with a sulfonamide group, it is possible to attain substrate activation by proton‐coupled electron transfer (PCET) [98, 99] without employing a Brønsted acid co‐catalyst. Despite the dual reference system (Scheme 17), combining complex Cu(dq)(BINAP)BF_4_ with diphenylphosphoric acid (p*K*_a_ ∼ 3.72 in DMSO) gave better results in terms of yield, combination with the much less acidic free pyrazole‐pyridine sulfonamide (p*K*_a_ ∼ 16.1) was found unable to promote the reaction. Thus, the observed activity of PC **22** can be confidently ascribed to the enforced proximity between the Brønsted acidic site and the photoactive unit (Scheme 17).

**SCHEME 18:**
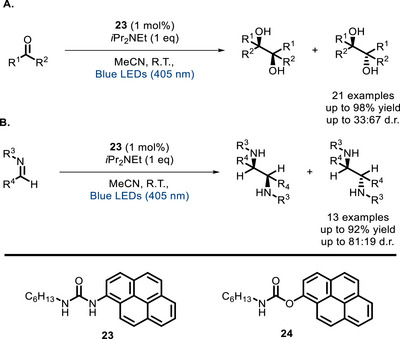
Pinacol coupling catalyzed by bifunctional pyrene derivatives.

Another interesting example of bifunctional PC, involving urea as a hydrogen bond‐donor, was described by Okamoto later in 2024 (Scheme 18) [100]. Covalent linkage between the pyrene moiety, acting as a photosensitizer, and the hydrogen‐bonding residue allowed to obtain significantly higher yields in the pinacol coupling compared to the two units alone and to a bifunctional analog featuring a carbamate (with only one hydrogen‐bonding moiety) instead of the urea group. Computational studies demonstrated that hydrogen bonding lowers the LUMO energy of substrate's C = O, and urea **23** exerts the strongest effect across the series pyrene/**23**/**24**. Moreover, PC **23** also displays the largest substrate‐catalyst intermolecular interaction energy across the series. These results are consistent with the observed ability to promote the reductive coupling.

**SCHEME 19:**
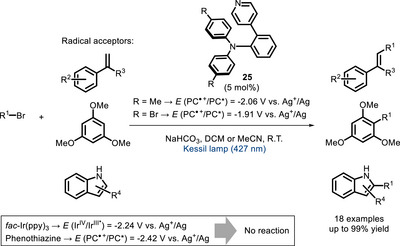
Activation of aryl bromides using the bivalent photocatalyst **25**. Potentials reported are measured using the couple Ag^+^/Ag (0.1 M AgNO_3_ + 0.1 M *n*Bu_4_NPF_6_) in MeCN.

In 2022, Kato, Nanjo, and Takemoto reported a metal‐free pyridine‐based donor‐acceptor system (**25**, Scheme 19) absorbing visible light [101]. The photoexcited form of **25** is able to generate alkyl radicals from alkyl bromides, exploiting a halogen bond interaction. This allows to react substrates which cannot be reduced by PCs, such as *fac*‐Ir(ppy)_3_ and phenothiazine, featuring a more negative potential *E* (PC^•+^/PC*) compared to **25**. The radical intermediates react with different acceptors in a C‐C coupling.

**SCHEME 20:**
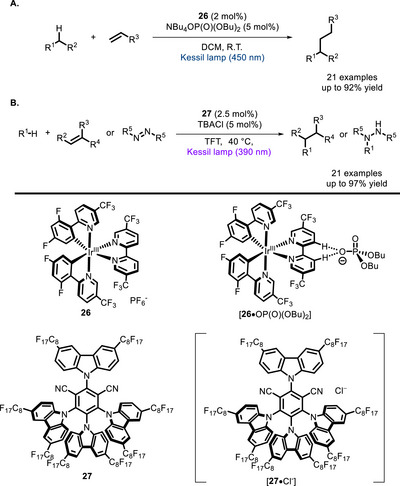
Supramolecular bifunctional photocatalysts catalyzing Giese‐type reactions by coordinating, respectively, a phosphate base (A) and a chloride anion (B).

Proximity between the catalytic units may be enforced not only by covalent linkage, but also by the formation of noncovalent interactions. For example, in 2019, the group of Knowles and Alexanian demonstrated that a phosphate base coordinated to the Ir^III^ complex **26** could be used as an HAT reactant in a Giese reaction [102]. This interaction, which was studied by ^1^H‐NMR titration, proved to be crucial to the reactivity observed: indeed, the oxidation of the phosphate base by the Ir^III^ complex is thermodynamically endergonic, but is facilitated by the binding between the two species (Scheme 20A).

In 2024, Vincent and co‐workers reported the beneficial effect of proximity in Giese‐type reactions catalyzed by the supramolecular complex [**27**•Cl^–^], which is made up of a fluorinated derivative of **4CzIPN** (**27**) in situ coordinated to Cl^–^ [103]. Upon absorption of light, Cl^–^ is then oxidized to Cl^•^, which is the catalytically active species. These reactions proceeded in good to excellent yields at catalyst loadings as low as 2.5 mol%, while the unfunctionalized **4CzIPN** was unreactive under the same conditions (Scheme 20B). This difference in performance was attributed to proximity, which makes oxidation of Cl^−^ easier.

**SCHEME 21:**
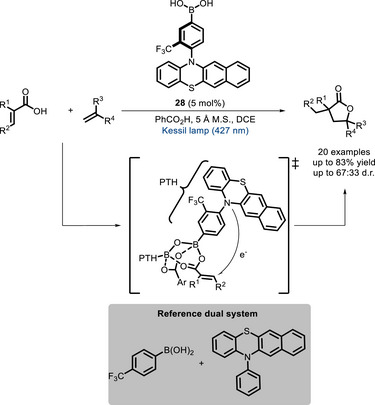
Cooperative catalysis for the synthesis of γ‐lactones through a [3+2] cycloaddition reaction between α,β‐unsaturated carboxylic acids and olefins.

A year later, Takemoto and co‐workers developed a bifunctional PC (**28**) featuring a boronic acid moiety covalently linked to a benzophenothiazine (PTH) chromophore (Scheme 21) [104]. This catalyst was successfully employed in the synthesis of γ‐lactones through a [3+2] cycloaddition reaction between α,β‐unsaturated carboxylic acids and olefins. The use of cooperative catalysis promotes efficiently the formation of α‐carboxy radicals from α,β‐unsaturated carboxylic acids, without the need for strong acids or reductants. The carboxylic acid substrate is seemingly maintained in proximity of the excited PC by interacting with the boronic acid moiety. Although the observed catalytic activity of PC **28** is similar to that of the corresponding reference dual catalyst (Scheme 21), the bifunctional system displays better chemoselectivity in favor of α,β‐unsaturated acids versus amides and esters. Moreover, the methodology covers a broad substrate scope, including delicate substrates such as nitrogen‐substituted olefins, that are unstable under highly acidic conditions.

**SCHEME 22:**
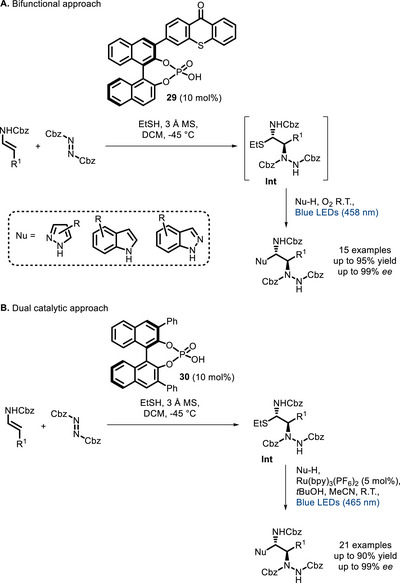
Sequential asymmetric vicinal difunctionalization of α‐unsubstituted enecarbamates exploiting, respectively, bifunctional PC **29** (A) and the dual catalytic system **30** / Ru(bpy)_3_(PF_6_)_2_ (B).

In 2020, Masson and co‐workers reported the successful use of several bifunctional chiral phosphoric acid(s) (see Scheme 22A for the most effective PC **29**) [105], bearing one or two thioxanthone photoactive unit(s), to improve the efficiency of a previously reported dual catalytic reaction (Scheme 22B) [106]. The latter transformation consists of the α‐amination of α‐unsubstituted enecarbamates followed by a Friedel‐Crafts‐type reaction with an N‐containing heterocycle (pyrazole, indazole, indole). The reaction is run in the presence of a thiol in order to generate the α‐carbamoylsulfide intermediate **Int** and minimize the formation of side products. In the first reported dual catalytic version (Scheme 22B) [106], combining phosphoric acid **30** and Ru(bpy)_3_(PF_6_)_2_, the reaction cannot be run in a one‐pot sequential fashion because different solvents (DCM and MeCN) must be used in the two steps, and purification of intermediate **Int** is required. The bifunctional approach exploiting PC **29** (Scheme 22 A) allowed to solve these issues, making the reaction entirely feasible in dichloromethane, without the need to isolate intermediate **Int**. Very high enantioselectivity was observed, along with improved yields compared to the dual system.

**FIGURE 3:**
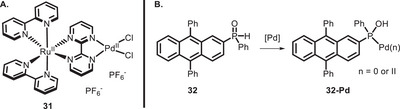
Pd‐binding bifunctional PCs reported by Mori‐Yamashita (A), and Nakajima‐Nemoto (B).

Switching to the realm of metal‐catalyzed reactions, among the first examples of a bifunctional PC useful for cross‐coupling, it is possible to mention the work of Mori, Yamashita, and co‐workers, involving a polymetallic Ru‐Pd complex (**31**) for the Suzuki‐Miyaura coupling (Figure 3A) [107]. While the reaction was also working in the dark, there was a significant improvement in the TON when the reaction mixture was irradiated, but the same improvement could not be seen when a Pd‐bipyridyl complex not absorbing light was used.

Later on, in 2022, Nemoto and co‐workers reported a diphenylanthracene functionalized with a secondary phosphine oxide (**32**) able to bind Pd (**32‐Pd**, Figure 3B) [108].

**SCHEME 23:**
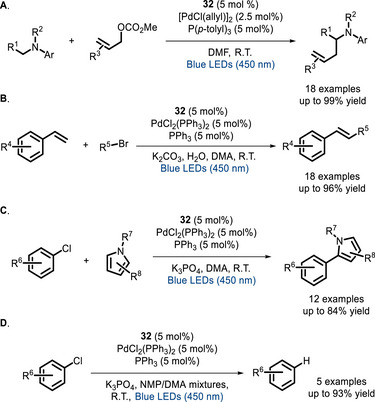
Allylation of alkyl amines (A), Heck reaction (B), biaryl synthesis (C), and dehalogenative hydrogenation (D) promoted by the in situ‐formed bifunctional PC **32**‐Pd.

The in situ‐formed catalyst **32‐Pd** was successfully applied to allylation of alkyl amines, Heck reaction, biaryl synthesis, and dehalogenative hydrogenation (Scheme 23), giving better yields compared to the dual system involving diphenylanthracene as PC and PPh_3_ as ligand for Pd.

**SCHEME 24:**
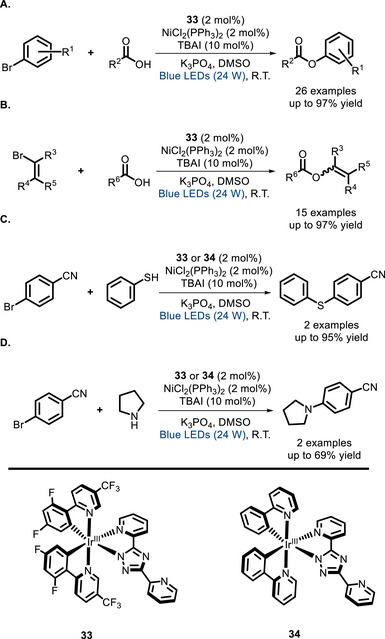
Bifunctional Ir^III^ PCs catalyzing C‐O (A and B), C‐S (C), and C‐N (D) couplings.

In 2022, the group of Lin developed a cooperative metallaphotoredox catalyst by incorporating into an Ir^III^ PC a series of different ligands for nickel [109]. Photophysical studies demonstrate that all these PCs express similar UV‐vis absorption and photoluminescence properties. The group demonstrated that an appropriate pendant binding site is crucial for the yields of the reaction ascribing these results to a different electron transfer mechanism and a better stability against photodecomposition. These bimetallic systems were also tested in C‐O, C‐S, and C‐N coupling reactions (Scheme 24), giving much better results in comparison with the corresponding bimolecular systems at low catalytic loading (down to 2 mol%).

**SCHEME 25:**
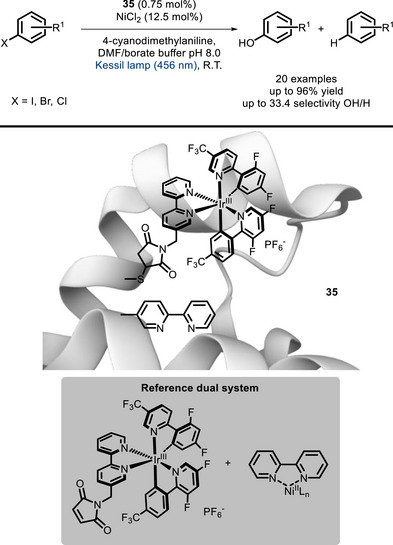
Artificially modified enzyme for the hydroxylation of different aryl halides.

Another advancement in the field was published in 2023 by Lee and Song, who reported an artificially modified enzyme (**35**) able to bind Ni and promote the metallaphotoredox‐catalytic synthesis of phenols from aryl halides [110]. By introducing noncanonical amino acids in a modified sperm whale myoglobin, it was possible to keep the dye (derived from an Ir^III^ complex) and the Ni^II^(bpy) catalytic moiety close together, obtaining formation of the desired phenol while minimizing the undesired dehalogenation product (Scheme 25). Indeed, the bifunctional PC **35** shows about five times higher selectivity (compared to the reference dual catalytic system) for the desired phenol product versus the dehalogenation product.

**SCHEME 26:**
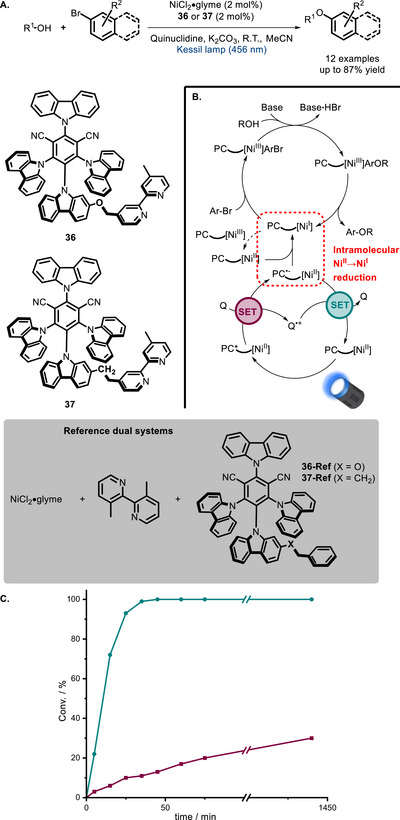
Bifunctional bipyridine‐derived PCs for the Ni‐mediated C‐O coupling between aliphatic alcohols and aryl bromides (A). Postulated catalytic cycle and role of proximity (B). Kinetic profile of the reaction between *n*‐hexanol and 4‐bromoacetophenone with the bifunctional setup [1:1 **36** / NiCl_2_(glyme)] (‐●‐) and the dual catalytic setup [1:1:1 **36‐Ref** / NiCl_2_(glyme) / 4,4’‐dmbpy] (‐■‐) at 0.5 mol% catalytic loading (C). Q = quinuclidine.

In 2024, our research group reported two bifunctional donor‐acceptor cyanoarenes (**36** and **37**) functionalized with a 2,2’‐bipyridine moiety [111]. The corresponding in situ‐formed Ni complexes were found to catalyze visible light‐promoted C‐O cross‐coupling at a much faster rate than the corresponding dual catalytic systems (Scheme 26A and C). As a consequence, the catalytic loading could be reduced to 0.5 mol% without significantly affecting the yield. Although these Ni‐photoredox C‐O couplings were originally designed as synergistic dual catalytic reactions (Figure 1A) [112], they were later shown to work with a relay mechanism (Scheme 26B): a dark Ni^I^/Ni^III^ catalytic cycle is sustained by a photocatalytic cycle continuously regenerating Ni^I^, which tends to rapidly decompose to the inactive Ni^II^ form [74, 113]. The superior performance of the bifunctional PCs compared to the dual systems is ascribed to a faster Ni^II^→Ni^I^ reduction operated by the radical anion PC^●−^, which becomes an *intramolecular step*.

**SCHEME 27:**
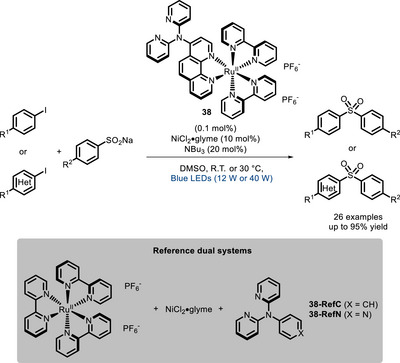
Coupling of iodides and sulfonates catalyzed by a bifunctional Ru^II^‐polypyridyl complex.

In the same year, Abel, Beletskaya, and co‐workers proved that the conjugation of a Ru^II^‐polypyridyl complex with a bipyridine (**38**) can significantly improve the yield in the coupling between sulfonates and aryl halides when compared to the corresponding dual catalytic system [114]. Moreover, the system was able to deliver the product in almost quantitative yields with catalyst loading as low as 0.1 mol% (Scheme 27).

**SCHEME 28:**
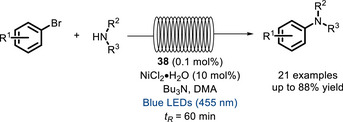
C‐N coupling in flow catalyzed by bifunctional Ru^II^‐polypyridyl complex **38**.

Later, the same catalysts were applied to the cross‐coupling of aryl bromides and amines [115]. In this case, while in the classical “batch” reaction, the dual approach was still the best performing, it was found that the bifunctional system gives the best results in flow conditions (Scheme 28).

**SCHEME 29:**
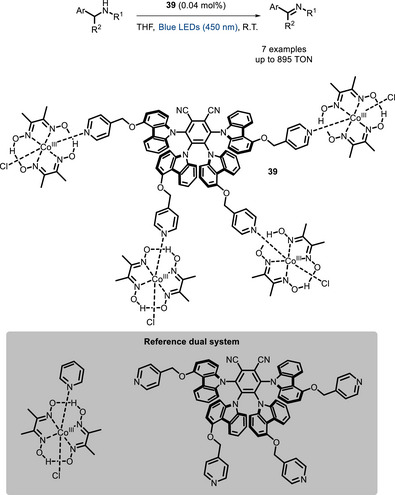
Dehydrogenation of secondary amines catalyzed by Co‐bifunctional PC **39**.

Although Ni‐photoredox catalysis is the most represented, bifunctional PCs involving the use of other metals have been developed as well. In 2019, Chao and Zhao reported the ability of a pyridine‐functionalized donor‐acceptor cyanoarene (**39**) to coordinate Co and catalyze the dehydrogenation of secondary amines [116]. It was found that this bifunctional PC has a sixfold higher TON than the mixture of the corresponding cyanoarene dye with a cobaloxime complex (Scheme 29). Bifunctional catalysts of this kind have also been extensively used for CO reduction in the past, and reviewed elsewhere [117], and hence will not be discussed in this work.

**FIGURE 4:**
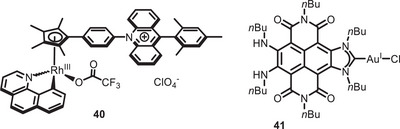
Bifunctional Rh^III^ and Au^I^ complexes.

Another example was reported later in 2020 by Chang and co‐workers, who synthesized a Rh^III^ catalyst covalently bound with an acridinium photosensitizer (b, Figure 4) [118]. Having established, by means of cyclic voltammetry and EPR measurements, that a metal‐to‐ligand charge‐transfer state is actually possible, the authors also showed the application in the aromatic C‐H arylation and methylation of benzoquinoline.

In 2024, Ruiz‐Zambrana, Poyatos, and Peris reported the photocatalytic use of a previously synthesized [119] naphthalene‐diimide‐functionalized N‐heterocyclic carbene‐Au^I^ complex (**41**, Figure 4) [120]. After determining the ability of this complex to catalyze the formation of singlet oxygen and let it react with different substrates (e.g., diphenyl naphthalene, 2‐chloroethyl ethyl sulfide) to form peroxides, they tested its ability to act as a bifunctional PC in the coupling between diazonium salts and trimethylsilylethynylbenzene or mesitylene. The reaction showed faster kinetics compared to the one involving a Ru^II^ photosensitizer and AuCl(PPh_3_) as a catalyst. In the proposed mechanism, indeed, there is a close cooperation between the Au center and the carbene moiety, involving a ligand‐to‐metal single electron transfer.

**SCHEME 30:**
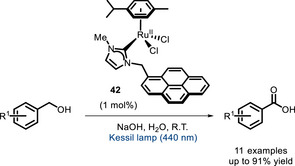
Oxidation of benzylic alcohols catalyzed by NHC‐Ru^II^ complex 42.

Finally, in 2025, an NHC‐Ru^II^‐complex (**42**) featuring a photoactive perylene moiety was reported by Ibáñez‐Ibáñez, Guisado‐Barrios, and Mata [121]. This catalyst was tested in the oxidation of benzylic alcohols to carboxylic acids (Scheme 30), and the light irradiation was found crucial in assisting the nucleophilic attack of water leading to oxidation. Analogous systems lacking a light‐absorbing moiety displayed, indeed, lower TONs.

## 3 Bivalent Photocatalysts

The bifunctional PCs presented in Section 2 consist of two (or more) well‐distinct catalytic units, including the photoactive group. In this section, instead, systems in which *a single catalytic unit can perform a double role* (e.g., generation of a photoexcited species capable of electron transfer and substrate coordination/activation) are discussed and classified as “bivalent photocatalysts”. Thus, cases of simple catalyst photoactivation (e.g., by photolysis of a ligand) are not discussed.

**SCHEME 31:**
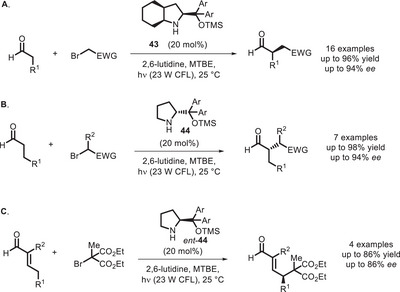
α‐Alkylation of aldehydes where light absorption is mediated by a colored EDA complex (A) or by the enamine itself (B and C). Ar = 3,5‐(CF_3_)_2_‐C_6_H_3_.

**SCHEME 32:**
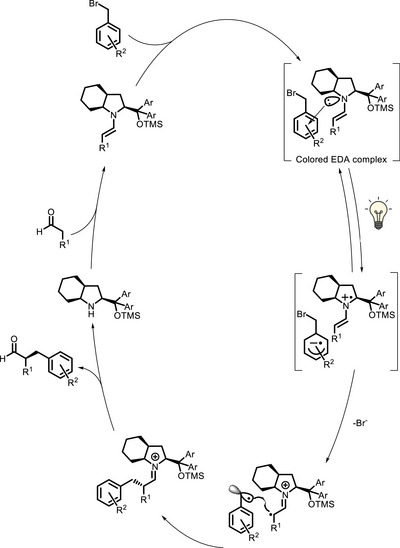
Catalytic cycle for the visible light‐promoted α‐alkylation of aldehydes involving formation of an EDA complex.

Primary and secondary amines can act as bivalent PCs because they can react with carbonyl compounds and generate enamine intermediates, which are photoactive and nucleophilic at the same time. Iconic examples of this reactivity have been reported, again in the α‐alkylation of aldehydes, by the Melchiorre group. A seminal paper published in 2013 reported that enamines are able to form colored EDA complexes with the electron‐poor aryl bromides (Scheme 31A) [92]. Upon visible light absorption, a bromide anion is lost from the photoexcited EDA complex with concurrent formation of a radical that reacts with the enamine/iminium species (Scheme 32). With the same approach, also cyclic ketones can be functionalized, in which case a primary amine must be employed as organocatalyst [122].

**SCHEME 33:**
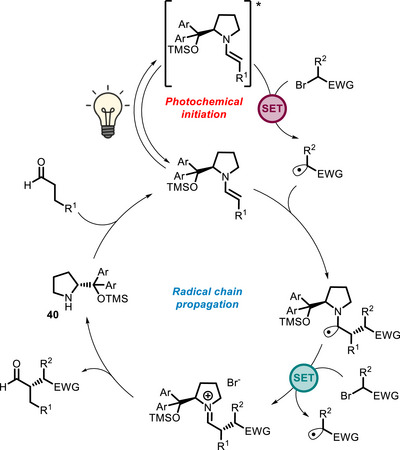
Catalytic cycle for the visible light‐promoted α‐alkylation of aldehydes initiated by oxidative quenching of the photoexcited enamine [93].

Later on, Melchiorre further developed this concept, showing that, even when no EDA complex can be formed (for example, when the electrophile is a bromomalonate), there is the possibility that the enamine itself absorbs light and initiates the enantioselective alkylation reaction (Scheme 31B) [93]. In this case, the cycle proposed involves, after photoinitiation, a chain propagation mechanism, where the α‐amino radical can act as a reducing agent with respect to the bromomalonate (Scheme 33). In the same publication, this approach could be successfully extended to α,β‐unsaturated aldehydes (Scheme 31C).

**SCHEME 34:**
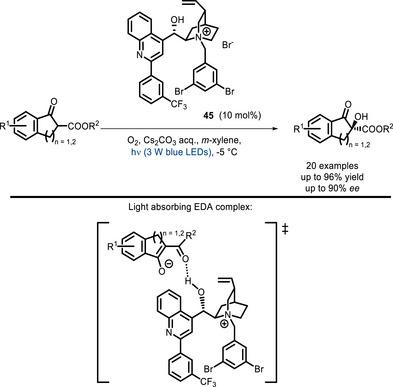
Aerobic oxidation of β‐ketoesters catalyzed by PTC catalyst **45**.

EDA complexes have been successfully used in several instances. For example, Meng and co‐workers reported the oxygenation of β‐dicarbonyl compounds with the cinchona‐alkaloid‐derived PTC **45** [123]. This is an extension of the above‐discussed approach relying on a cinchona‐derived PTC conjugated to a porphyrin (Scheme 14). Here, the absorbing species is the chiral EDA complex formed between a stabilized enolate and the PTC, which can be quenched by ^3^O_2_ to produce the reactive ^1^O_2_ form (Scheme 34).

**SCHEME 35:**
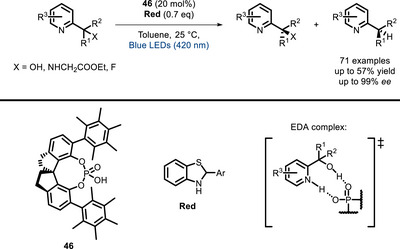
Kinetic resolution of alcohols, amines, and fluorides catalyzed by EDA complexes with chiral phosphoric acid 46.

A very recent example consists of the kinetic resolution of azaarene‐functionalized tertiary alcohols, amines, fluorides, and ethylene oxides with chiral phosphoric acids (**46**, Scheme 35) [124]. The formation of the EDA complex is indeed favored with one enantiomer, and after absorption of light, it can undergo single‐electron transfer with a reductant (the authors used a thiazole derivative), leading to deoxygenation, deamination, or defluorination, respectively.

**SCHEME 36:**
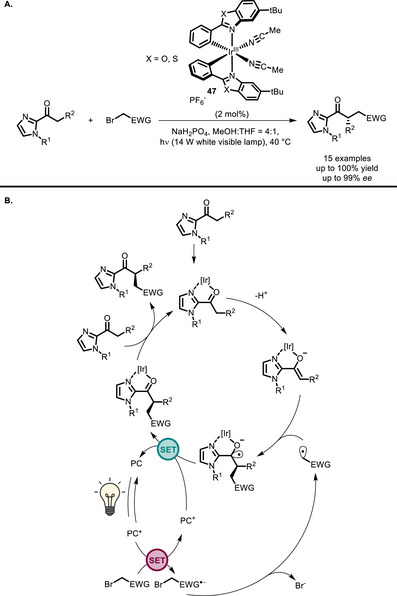
α‐Alkylation of acyl imidazoles catalyzed by Ir^III^ complex **47** (A), and the corresponding catalytic cycle (B).

In 2014, Meggers and co‐workers developed the chiral Ir^III^ photocatalyst **47** for the enantioselective alkylation of 2‐acyl imidazoles (Scheme 36) [125]. This example is particularly interesting, since the Ir^III^ complex itself is able to act at the same time as photocatalyst and as an organometallic catalyst: indeed, the acetonitrile ligands are displaced by the reaction substrate, which can chelate Ir, and is thus kept in the chiral environment created by the helical complex. The electron‐poor alkylating agents (benzyl or phenacyl bromides) oxidatively quench PC*, generating radical species which are intercepted by the substrate's enolate.

**SCHEME 37:**
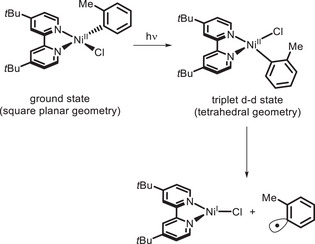
Photoinduced formation of Ni^I^ species.

In the same years, Doyle and colleagues published a report showing that Ni^II^ bipyridyl complexes themselves could absorb light to generate a Ni^I^ species [126, 127], which can enter the Ni^I^/Ni^III^ catalytic cycle [113, 128] delivering the C‐O coupling product. Indeed, the square planar Ni^II^ complex reaches an MLCT state rapidly decaying into a ^3^(d‐d) state with tetrahedral geometry, from which Ni^II^‐Ar bond homolysis can occur (Scheme 37).

**SCHEME 38:**
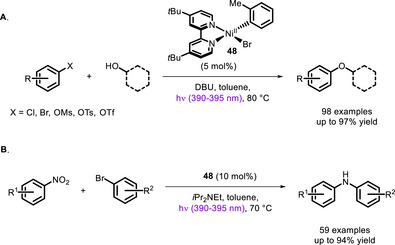
C‐O (A) and C‐N (B) coupling catalyzed by a Ni^II^ complex in the absence of any external photosensitizer.

This approach was then exploited by Xue and colleagues to develop a protocol to carry out C‐O and C‐N cross‐couplings using near‐UV light (390–395 nm), without the need to add an external PC (Scheme 38) [129, 130]. In both cases, the wavelength of irradiation turned out to be crucial, since the use of either longer or shorter wavelengths led to a drop in yields. While not being strictly an example of bivalent photocatalysis as defined above (indeed, light is needed to continuously regenerate the PC), the work reported by Doyle and Xue paved the way for the development of other bivalent or bifunctional ligands.

**SCHEME 39:**
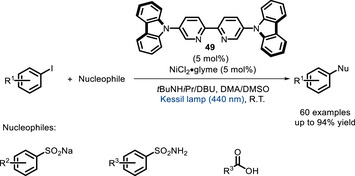
Bifunctional bipyridine ligand 49 exploiting intraligand charge transfer.

Indeed, shortly after these contributions, van der Veen, Thomas, Pieber, and co‐workers developed a carbazole‐functionalized bpy ligand (**49**, Scheme 39) whose in situ‐prepared Ni^II^ complex can absorb visible light and generate a Ni^I^ catalyst for C‐S, C‐O, and C‐N cross‐couplings [131]. Upon irradiation, the complex reaches an excited state characterized by intraligand charge transfer (ILCT), which is similar to the excited state observed with D‐A cyanoarenes [132]. This state then decays into a metal‐centered ^3^(d‐d) state, from which Ni‐Cl bond homolysis can occur as shown in Scheme 37, generating an active Ni^I^ species. The substrate scope was quite limited on the nucleophile side, with only sulfinates, carboxylic acids, and sulfonamides being reactive, while amines and thiols did not afford the desired coupling product (Scheme 39).

**SCHEME 40:**
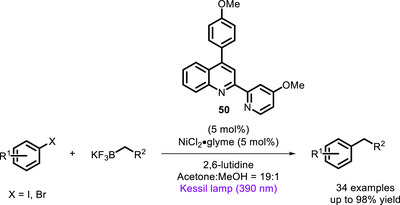
Ni‐mediated coupling featuring bivalent ligand **50**.

Almost at the same time, Li and co‐workers applied the same concept to develop a bivalent PC (**50**, Scheme 40), which was successfully applied in the Ni‐mediated coupling of aryl iodides and bromides with trifluoroborates [133]. Interestingly, the same complex proved to be effective in different other reactions (such as C‐N, C‐O, C‐P coupling), as well as in reactivities implying metals different from Ni (Fe, Co, Cu).

**SCHEME 41:**
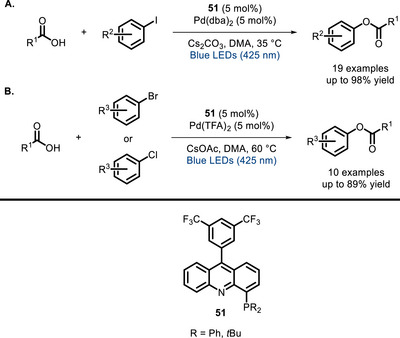
Cross‐coupling between aryl iodides (A) or bromides/chlorides (B) and carboxylic acids catalyzed by bifunctional Pd‐acridine ligand **51**.

Iwasawa and co‐workers applied the ‘bivalent PC’ concept to palladium catalysis, developing an acridine ligand (**51**) whose in situ‐formed Pd complex promotes the coupling between aryl halides and carboxylic acids (Scheme 41). [134]. In this case, light absorption facilitates the otherwise sluggish Pd^II^→Pd^0^ reductive elimination, yielding the coupling product.

## 4 Summary and Outlook

In this review, we have highlighted several recent examples demonstrating the benefits of bifunctional PCs, including cases in which two distinct functions are embedded within the same functional group (“bivalent PCs”). While research effort in this area has long been driven by the pursuit of enantioselectivity, in recent years, bifunctional PCs have also been explored as a strategy to obtain globally enhanced catalytic performance compared to dual catalytic systems. Proximity between catalytic groups (or their integration into a single motif) may allow to more effectively exploit the short‐lived intermediates typical of photocatalytic reactions, thereby leading to higher activity and reduced catalytic loading. Unfortunately, not in all contributions means are provided to clearly assess this effect by comparison with a dual catalytic reference system. Moreover, the improvement obtained is often not sufficient to justify the additional synthetic effort and the reduced operational flexibility compared to dual catalytic systems. Despite these cautions, the examples discussed clearly indicate the potential of the bifunctional approach, which can enable more effective synthetic methodologies and even disclose new types of reactivity. Overall, we believe that research in this fascinating area is still in its early stages and holds considerable promise for the future, provided that some challenges are met, such as: i) developing recyclable systems, which can be recovered/reused or immobilized onto a solid support, in order capitalize the synthetic effort required for their preparation; ii) employing long wavelength‐absorbing photoactive units in order to exploit the advantages of red/near IR light (easier scalability, broader functional group tolerance) while overcoming its inherent limitations (narrow range of redox potentials/ low excited state energies).

## Conflicts of Interest

The authors declare no conflicts of interest.
